# Non-Aqueous Glycerol Monolaurate Gel Exhibits Antibacterial and Anti-Biofilm Activity against Gram-Positive and Gram-Negative Pathogens

**DOI:** 10.1371/journal.pone.0120280

**Published:** 2015-03-23

**Authors:** Elizabeth A. Mueller, Patrick M. Schlievert

**Affiliations:** Department of Microbiology, Carver College of Medicine, University of Iowa, Iowa City, IA 52242, United States of America; The Scripps Research Institute and Sorrento Therapeutics, Inc., UNITED STATES

## Abstract

**Background:**

Skin and surgical infections due to *Staphylococcus aureus*, *Pseudomonas aeruginosa*, and *Acinetobacter baumannii* are causes of patient morbidity and increased healthcare costs. These organisms grow planktonically and as biofilms, and many strains exhibit antibiotic resistance. This study examines the antibacterial and anti-biofilm activity of glycerol monolaurate (GML), as solubilized in a non-aqueous vehicle (5% GML Gel), as a novel, broadly-active topical antimicrobial. The FDA has designated GML as generally recognized as safe for human use, and the compound is commonly used in the cosmetic and food industries.

**Methods:**

In vitro, bacterial strains in broths and biofilms were exposed to GML Gel, and effects on bacterial colony-forming units (CFUs) were assessed. In vivo,subcutaneous incisions were made in New Zealand white rabbits; the incisions were closed with four sutures. Bacterial strains were painted onto the incision sites, and then GML Gel or placebo was liberally applied to cover the sites completely. Rabbits were allowed to awaken and were examined for CFUs as a function of exposure time.

**Results:**

In vitro, GML Gel was bactericidal for all broth culture and biofilm organisms in <1 hour and <4 hour, respectively; no CFUs were detected after the entire 24 h test period. In vivo, GML Gel inhibited bacterial growth in the surgical incision sites, compared to no growth inhibition in controls. GML Gel significantly reduced inflammation, as viewed by lack of redness in and below the incision sites.

**Conclusions:**

Our findings suggest that 5% GML Gel is useful as a potent topical antibacterial and anti-inflammatory agent for prevention of infections.

## Introduction

Although the skin is a prominent physical and immunological barrier, some bacterial pathogens cause a variety of skin and soft tissue infections (SSTIs), including impetigo, boils, and cellulitis. Surgical site infections (SSIs) are among the most serious SSTIs and are associated with substantial health and economic costs, accounting for 14–17% of all hospital-associated infections (HAIs) and for 38% of HAIs of surgical patients [1, 2]. Patients acquiring SSIs are more likely to have increased lengths of hospitalization, increased odds of readmission, and greater likelihood of admission to intensive care units (ICUs) [3]. Importantly, these patients are twice as likely as non-infected patients to succumb during hospitalization [3]. These factors not only adversely contribute to patient morbidity and mortality, they also lead to SSIs costing an estimated $1.6 billion in U.S. hospital charges annually [4].

*Staphylococcus aureus* is the leading cause of SSIs (30%) [5]. Although a common human commensal, with an average of 40% of the population colonized intranasally at any given time, the organism produces a myriad of cell surface and secreted virulence factors, which make it a formidable pathogen [6]. Furthermore, antibiotic resistant strains have rapidly emerged, the most common of which is methicillin-resistant *S*. *aureus* (MRSA). MRSAs account for over 50% of hospital-associated *S*. *aureus* infections and have been associated with lower frequency of primary healing and delayed healing among SSIs [7, 8]. Even among methicillin-susceptible *S*. *aureus* (MSSA), many isolates are capable of forming persistent biofilms, which have low rates of metabolism and are often impenetrable by antibiotics [9].

In addition to *S*. *aureus*, important nosocomial Gram-negative pathogens are routinely causative of SSIs, including *Pseudomonas aeruginosa* (5.6%) and *Acinetobacter baumannii* (0.6%) [5]. Like *S*. *aureus*, both organisms produce a diverse range virulence factors, are capable of forming biofilms, and are increasingly acquiring antibiotic resistance [5, 10, 11]. The broad range of resistant bacterial isolates provides a serious public health concern unless novel antimicrobials can be developed.

Glycerol monolaurate (GML) (2,3-dihydroxypropyl dodecanoate) is a fatty acid monoester that is recognized as a safe, natural compound by the FDA and is commonly used as an emulsifier and preservative in the cosmetic and food industries. It has also been recognized as a broad-spectrum antibacterial agent, particularly against gram-positive pathogens, including gram-positive cocci, *Bacillus anthracis*, and clostridia by interfering with surface signal transduction systems [12, 13]. However, the compound likely has multiple mechanisms of action. GML inhibits gram-positive exotoxin production and removes pre-formed *S*. *aureus* biofilms at sub-growth-inhibitory concentrations [12–14]. Additionally, it has been documented to have anti-inflammatory activity in tissue culture and *in vivo* at host mucosal surfaces, which reduces infection establishment by gram-positive bacteria and enveloped viruses, including simian immunodeficiency virus (SIV), without altering the normal microflora [15–18]. Combined, these properties make GML a potential candidate as a safe tropical microbicide.

GML exhibits limited effectiveness against gram-negative organisms, principally those with an intact lipopolysaccharide (LPS) layer, including many pathogenic *Gammaproteobacteria* and *Enterbacteriaceae* [13]. Disruption of the LPS increases GML antibacterial activity; although resistant to GML natively, an isogenic *Salmonella enterica* serovar Minnesota Re mutant, that lacks the LPS O side chain, is susceptible to killing by GML. Similarly, co-treatment with GML and LPS-disrupting agents, including ethylene diamine tetraacetic acid (EDTA), significantly increases GML killing of gram-negative organisms [13]. However, due to GML solubility constraints, it is unlikely that GML will be suspended in aqueous solutions with EDTA for clinical application.

Preliminary studies indicate that a biocompatible, non-aqueous (NA) delivery vehicle may also have membrane-disrupting effects against bacterial pathogens and therefore, may be a clinically useful solubilizing agent and accelerant (synergizer) with GML [13]. In addition to its activity as a general microbial disruptant, the NA vehicle increases GML solubility by over 100-fold, allowing higher doses of GML to be delivered topically against microbes. Combined, these properties suggest that a GML NA Gel may have broader antimicrobial activity than GML alone, and this might include gram-negative organisms with intact LPS. In the present study, we synthesized a 5% GML NA Gel and assessed its antibacterial and anti-biofilm activity *in vitro* and in an *in vivo* surgical site infection model.

## Methods

### Ethics

Rabbit experimentation was performed by us as approved by University of Iowa IACUC protocol 1106140 and University of Minnesota IACUC protocol number 0610A95086. All animals were anesthetized with ketamine and xylazine, and all received buprenorphine for pain management during experimentation.

### Non-aqueous gel (NA) and 5% GML Gel synthesis

Propylene glycol (Gallipot, St. Paul, MN; 73.55% w/w) was mixed with polyethylene glycol (Gallipot; 25% w/w) and hydroxypropyl cellulose (Gallipot; 1.25% w/w). The compounds were heated to 65°C for mixing and solubilization of GML, if added. GML (Colonial Chemical Inc, South Pittsburg, TN) was added to the non-aqueous vehicle 5% w/w to form the 5% GML Gel; this corresponds to a GML concentration of 50 mg/ml.

### Bacteria

*Staphylococcus aureus* MN8 is a typical menstrual TSS organism. The organism is classified as a pulsed-filed gel electrophoresis clonal type CDC USA200 organism; it is methicillin-sensitive and produces TSST-1. *Staphylococcus aureus* USA300 (LAC) is a community associated MRSA. *Klebsiella pneumoniae* Top52, a cystitis isolate, and *Escherichia coli* 25912 were provided by Dr. Steven Clegg, University of Iowa. *Pseudomonas aeruginosa* PAK, a widely studied strain know to express a full repertoire of virulence factors, was provided by Dr. Timothy Yahr at the University of Iowa. *Acinetobacter baumannii* was provided by Dr. Alexander Horswill, University of Iowa. All strains are maintained in the Schlievert laboratory as frozen stock cultures of low passage.

### Broth cultures

Bacteria were grown in Todd Hewitt broth (Difco Laboratories, Detroit, MI) overnight at 37°C in a standard incubator with aeration (shaking at 225 revolutions/min). Initial innocula were estimated by measuring absorbance at 600 nm wavelength and were verified by plate counts. For GML-treated planktonic cultures, bacteria were inoculated in 3 ml of media or 10% non-aqueous delivery vehicle solubilized in media in glass tubes to give an inoculum of 1 x 10^6^ CFU/ml. GML or ethanol vehicle control was then introduced to cultures. Cultures were shaken at 37°C for 24 h, at which point they were diluted for plate counts.

To assess effect of GML Gel on bacteria, 0.3 ml volumes of approximately 2 x 10^8^ CFU were added to 2.7 ml of 5% GML Gel and were mixed vigorously with sterile swabs. At desired time points, a swab of culture, containing approximately 0.1 ml volume of Gel was taken and spread on trypticase soy agar with 5% sheep blood agar (BD Biosciences, San Jose, CA).

### Biofilm cultures

Stationary biofilms in microtiter plates were formed as previously described [19]. Briefly, for GML-treated biofilms, 2 x 10^7^ bacteria in a medium that supports optimal biofilm growth were added to 96-well microtiter plate (Corning Inc, Corning, NY). *S*. *aureus* biofilms were cultured in tryptic soy broth (Difco) supplemented with 1% glucose, *P*. *aeruginosa* biofilms were cultured in Luria Broth (Difco), and *A*. *baumannii* biofilms were cultured in Todd Hewitt. Plates were incubated stationary at 37°C in the presence of 7% CO_2_ for up to 48 h with media changed at 24 h. Upon inoculation (for biofilm inhibition assays) or after maturation (for biofilm removal assays), biofilms were treated with sub-growth-inhibitory concentrations of GML or ethanol vehicle control. All conditions were tested at least in triplicate. At desired time points after GML treatment, media were removed and wells were washed three times with PBS. *S*. *aureus* biofilms were fixed with ethanol after washing. All biofilms were stained with 0.1% crystal violet for one hour and then washed three times with PBS. Remaining bound crystal violet was solubilized with 33% acetic acid for *S*. *aureus* and 95% ethanol for *P*. *aeruginosa* and *A*. *baumannii* and diluted up to 1:4. Absorbance was determined at 595 nm. If absorbance was out of range at 595, it was corrected to 605 or 610 nm.

For GML Gel-treated biofilms, 1 ml of 1 x 10^7^ bacteria were cultured in 24-well microtiter plates (Corning) for 48 h at 37°C in 7% CO_2_ and media was changed at 24 h. After maturation, media was removed, and 1 ml of 5% GML gel or non-aqueous vehicle was added to wells. At designated time points, wells were mixed vigorously for 30 seconds with sterile swabs that absorb approximately 0.1 ml of liquid culture and plated on trypticase soy agar with 5% sheep blood.

### Surgical site infection model

Animals were anesthetized with ketamine (10 mg/kg) and xylazine (10 mg/kg), and all received buprenorphine (0.05 mg/kg twice daily) for pain management during experimentation. Four centimeter subcutaneous incisions were made in the left flanks of three anesthetized New Zealand white rabbits per group and closed with four silk sutures. Then, 0.1 ml of approximately 10^10^ CFU of *S*. *aureus*, *P*. *aeruginosa*, or *A*. *baumannii* was inoculated onto the sutures. After five-minute’s incubation, the surgical sites were painted with 5% GML Gel or PBS control with sterile swabs. Rabbits were allowed to wake and surgical sites were examined for CFU counts with 0.1ml swabs at desired time points. The awakened animals did not show signs of pain or distress, but all animals were treated with buprenorphine (0.05 mg/kg) for pain relief. No animals required premature euthanasia, as defined by criteria established by the University of Iowa and University of Minnesota IACUCs. Thus, all animals were sacrificed at the end of experimentation with use of Beuthanasia D, and approved euthanasia agent. The surgical sites were then opened to examine inflammation of the underlying tissue visually.

### Statistics

Means and standard deviations were determined for all experiments. In studies to compare means between treatment groups, Student's *t* test analysis was used to determine significant differences.

## Results

### GML exhibits antibacterial and anti-biofilm activity against *Staphylococcus aureus*

**Fig 1:**
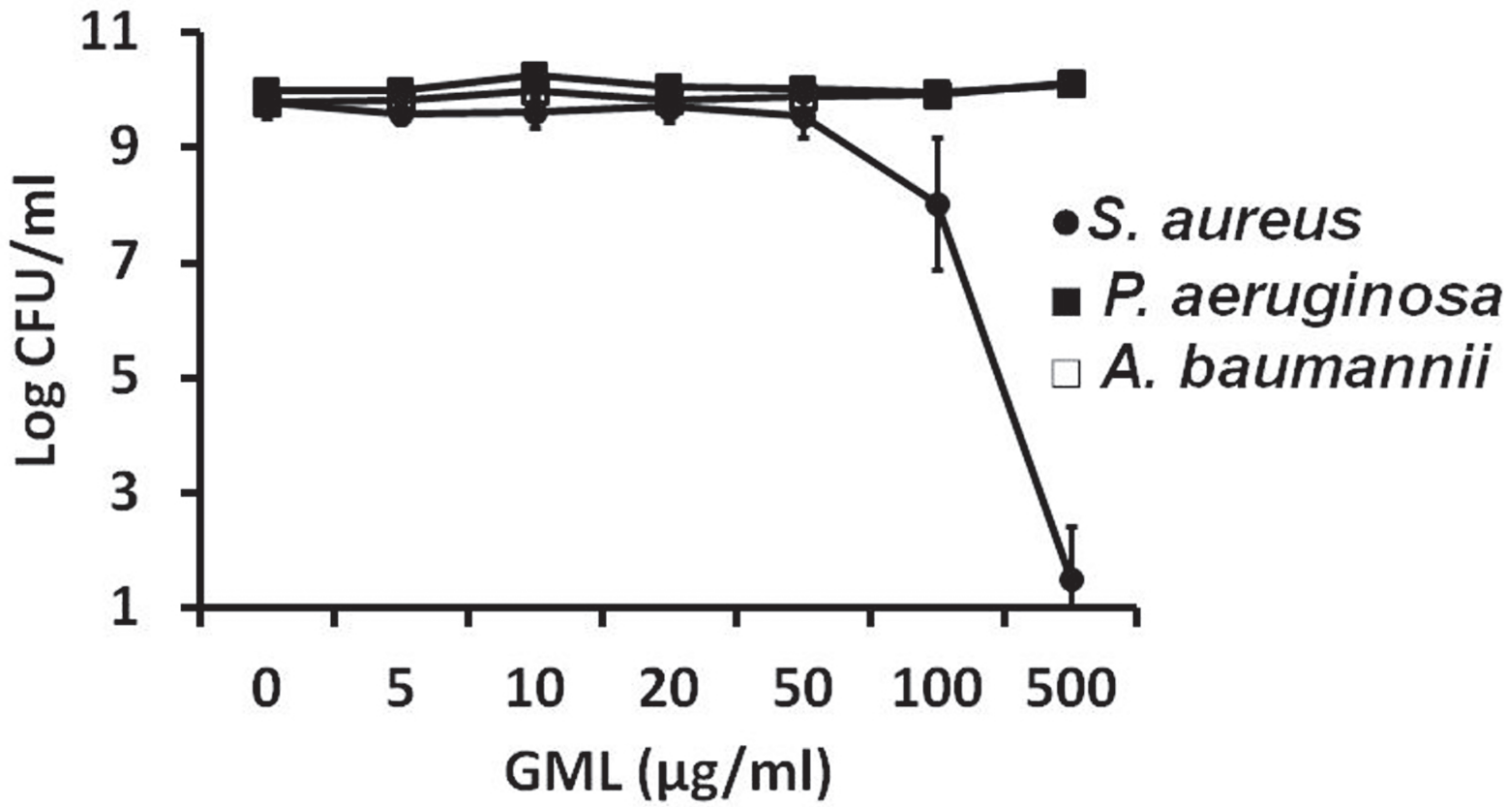
Activity of glycerol monolaurate against *Staphylococcus aureus*, *Pseudomonas aeruginosa*, and Acinetobacter *baumannii*. Glycerol monolaurate was incubated with 1 x 10^6^ CFU/ml of *Staphylococcus aureus*, *Pseudomonas aeruginosa*, and *Acinetobacter baumannii* for 24 h at 37°C at high aeration (shaking, 225 revolutions/min). Plate counts were used to determine CFU/ml. Bars show standard deviation among three replicates.

We compared the efficacy of the 5% NA GML Gel to GML alone, assessed against three bacterial strains. First, we exposed planktonic cultures of approximately 1 x 10^6^/ml to various concentrations of GML and assessed viability by plate counts 24 h post-treatment. Consistent with previously published data [13], GML was bactericidal (>3 log reduction in viable counts) to *S*. *aureus* MN8 at 500 μg/ml. However as expected, the compound did not exhibit antimicrobial activity for *P*. *aeruginosa* and *A*. *baumannii* at any concentration tested (up to 500 μg/ml) (Fig. 1).

**Fig 2:**
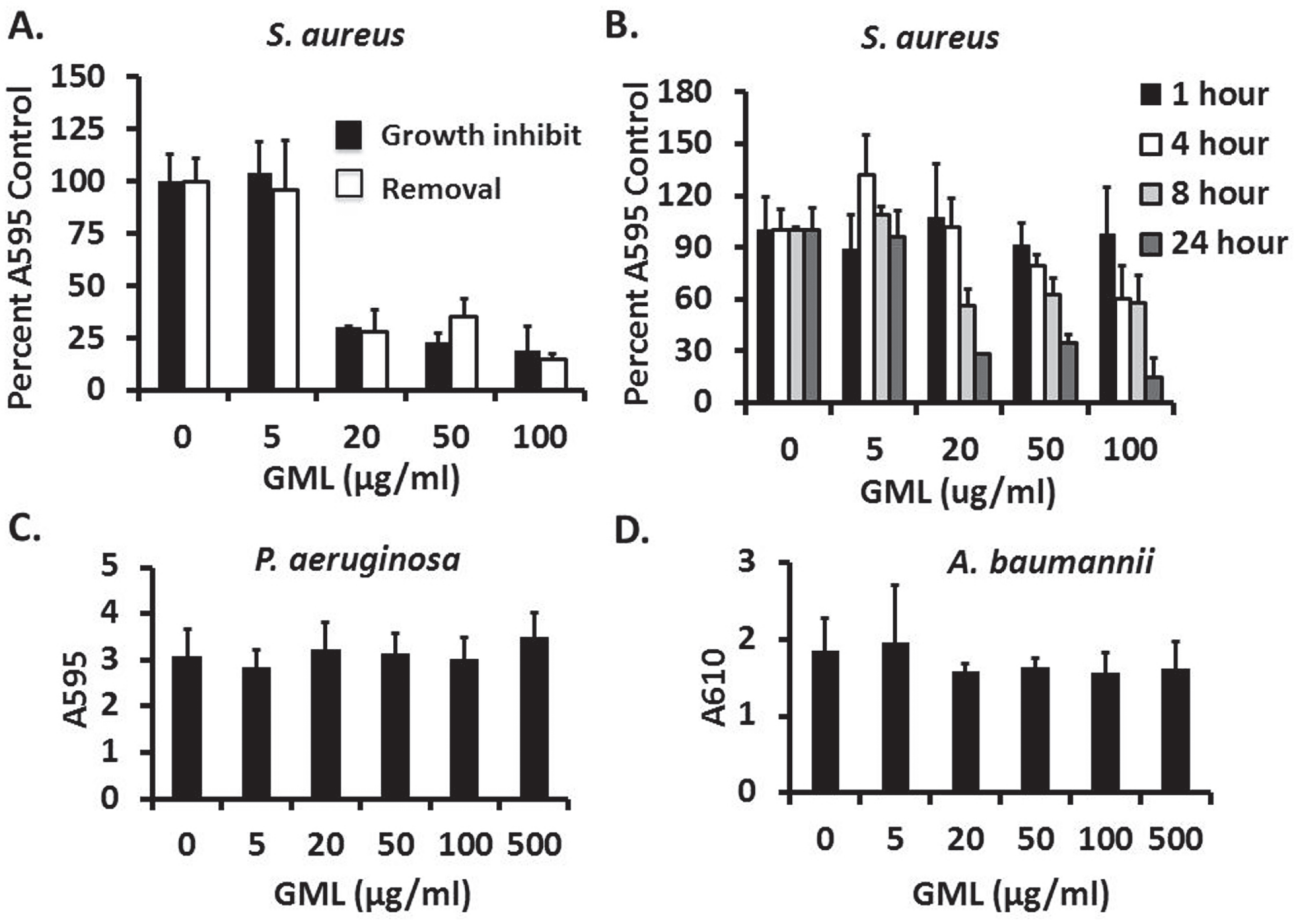
Anti-biofilm activity of glycerol monolaurate against *Staphylococcus aureus*, *Pseudomonas aeruginosa*, and *Acinetobacter baumannii*. Stationary biofilms were cultured in 96-well microtiter plates and treated with sub-growth-inhibitory concentrations of glycerol monolaurate. Twenty-four hours post-exposure, media was removed, wells were washed 3 times with PBS, biofilms were fixed with ethanol (*Staphylococcus aureus* biofilms only), and wells were treated with crystal violet. Wells were then washed to remove unbound crystal violet. Remaining crystal violet was solubilized with 33% acetic acid (*Staphylococcus aureus*) or 95% ethanol (*Pseudomonas aeruginosa* or *Acinetobacter baumannii*) and absorbance was determined by Tecan reader. Pre-formed and forming *Staphylococcus aureus* biofilms treated with sub-growth-inhibitory concentration of glycerol monolaurate and assessed for effect on biomass removal at 24 h post-treatment (A) and across time (B). Pre-formed *Pseudomonas aeruginosa* and *Acinetobacter baumannii* were treated with glycerol monolaurate and assessed for biomass remaining at 24 h post-treatment (C,D respectively). Bars represent standard deviation of at least three independent replicates.

To assess GML’s anti-biofilm activity, we treated either forming biofilms or mature stationary biofilms in microtiter plates with a range of sub-growth-inhibitory concentrations of GML. After incubation, planktonic cells were washed from the wells, and the wells were stained with crystal violet to measure remaining biomass. Of the three organisms tested, only *S*. *aureus* MN8 biofilms were both inhibited from growing and removed by treatment with GML (Fig. 2A). Treatment, with GML concentrations in excess of 12 μg/ml for 24 h, routinely removed 60–80% of the biomass compared to vehicle-treated controls. When treated with 100 μg/ml GML, the level of removable biofilm was comparable to 24 h treatment with 1 mg/ml proteinase K, which has been shown to remove *S*. *aureus* biofilms completely by disintegrating the proteinaceous biofilm matrix [20, 21]. Biofilm removal by GML was time-dependent, with higher concentrations acting at earlier time points (Fig. 2B). Unlike the staphylococcal biofilms, neither *P*. *aeruginosa* nor *A*. *baumannii* biofilms was removed by GML at any concentration tested (up to 500 μg/ml) (Fig. 2C,D).

### GML and NA vehicle synergize

**Fig 3:**
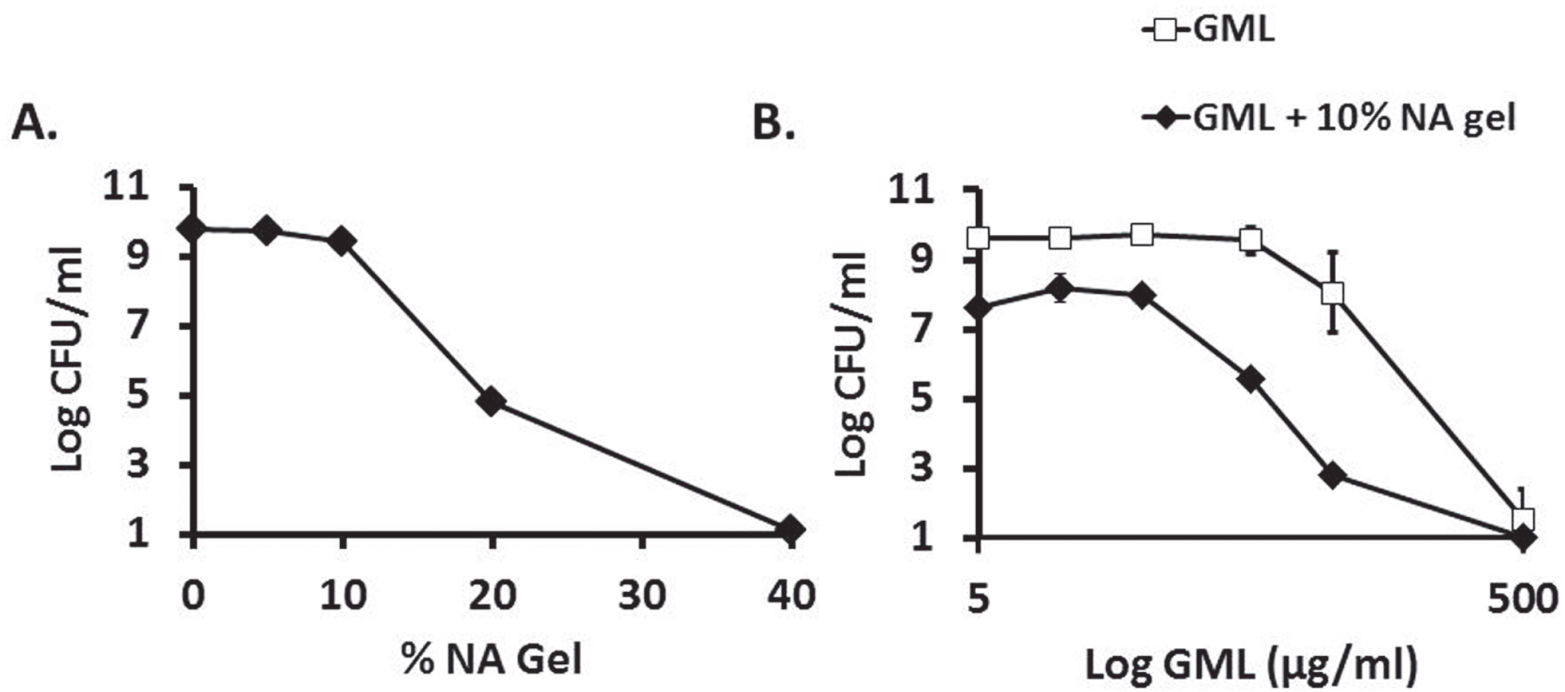
Antibacterial activity of non-aqueous gel alone and in combination with glycerol monolaurate against *Staphylococcus aureus*. Non-aqueous gel (v/v) was incubated with 1 x 10^6^ CFU/ml *Staphylococcus aureus* for 24 h at 37°C at high aeration (shaking, 225 revolutions/min). Plate counts were used to determine CFU/ml, and error bars represent standard deviation of three replicates (A). Sub-growth-inhibitory concentration of non-aqueous gel was also delivered with glycerol monolaurate and bacteria, and CFU/ml was likewise determined (B).

To obtain a baseline level of activity, we tested the NA vehicle for intrinsic antibacterial activity against *S*. *aureus* MN8. The vehicle was solubilized into broth medium at various percentages (v/v), inoculated with bacteria, shaken with high aeration for 24 h, and then assessed for viable organisms. At concentrations of ≤10% in broth, the NA gel allowed for bacterial growth to comparable levels as controls without NA. However, at concentrations exceeding 10%, the vehicle was bacteriostatic (20% NA) or bactericidal (≥40% NA) (Fig. 3A).

Using a sub-growth-inhibitory concentration of NA gel, we assessed for synergy between the vehicle and GML diluted in broth media against *S*. *aureus* MN8. Various concentrations of GML were added to 10% NA gel in broth and we conducted a standard dose-response. Statistically significant synergy (p ≤ 0.001) between compounds was seen at the lowest concentration of GML tested (5 μg/ml) compared to no significant growth defect for the ethanol vehicle control in combination with the NA vehicle. In the presence of the NA vehicle, a 5-times lower concentration of GML was bactericidal to MN8 (Fig. 3B).

Initially, we hypothesized that the NA vehicle and GML would synergize to remove biofilms, as the gel acts as a membrane disrupting agent, and therefore might be able to promote biofilm dissolution [13]. However, in our model, we were unable to assess the anti-biofilm activity of the NA gel independent of bacterial viability due to inability to wash the gel from the microtiter plate wells completely, even at low concentrations of NA (1%). Thus, for the remainder of the study, biofilm cultures treated with GML Gel or NA Gel will be examined for decrease in bacterial burden instead of biomass removal.

### GML Gel sterilizes planktonic and biofilm cultures of gram-positive and gram-negative pathogens

For clinical application, it is likely GML will be solubilized in the NA gel. Therefore, we synthesized a 5% (w/w) GML Gel and tested its activity against broth and biofilm cultures.

**Fig 4:**
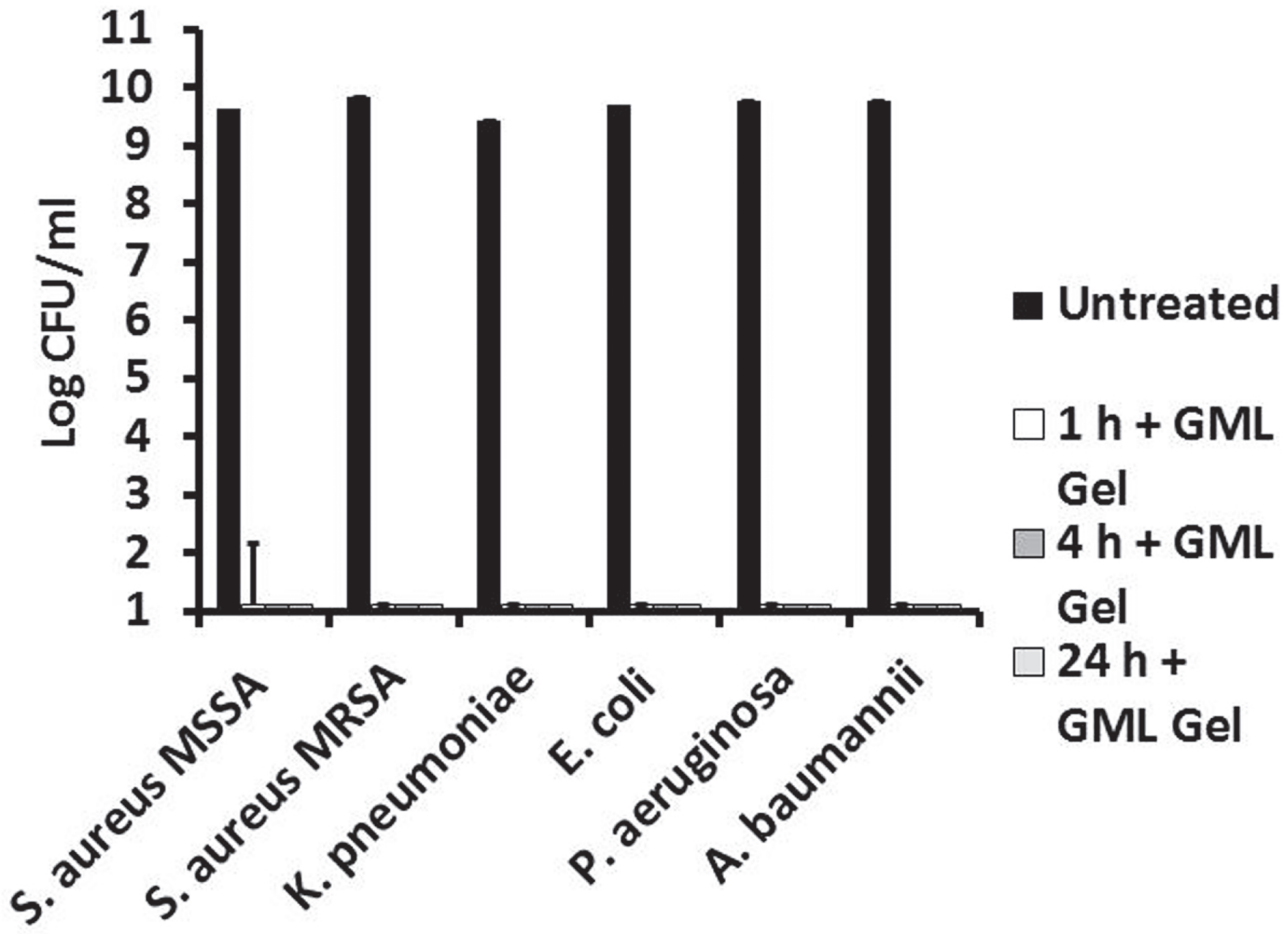
Antibacterial activity of 5% Glycerol Monolaurate Gel against gram-positive and gram-negative pathogens. 2.7 ml of 5% Glycerol Monolaurate Gel (w/w) was added to 0.3 ml of approximately 2 x 10^8^ organism in triplicate. Bacteria tested included a methicillin-susceptible *Staphylococcus aureus*, a methicillin-resistant *Staphylococcus aureus*, *Pseudomonas aeruginosa*, *Acinetobacter baumannii*, *Escherichia coli*, *and Klebsiella pneumoniae*. Cultures were mixed vigorously, incubated stationary in 7% CO_2_ at 37°C, and plated for CFU/ml. Bars represent standard deviation.

First, we exposed high inocula (2 x 10^8^ colony-forming units; CFUs) of bacterial cultures to the 5% GML. In addition to *S*. *aureus* MN8, *P*. *aeruginosa*, and *A*. *baumannii*, we also tested *S*. *aureus* USA300 LAC, a community-associated methicillin-resistant *S*. *aureus* (MRSA), and two gram-negative *Enterobacteriaceae*, *Klebsiella pneumoniae* and *Escherichia coli*. At various time points, cultures were mixed into GML Gel with sterile swabs, and 0.1 ml was plated in triplicate to determine survival. At one-hour post exposure to the 5% GML Gel, all cultures were completely or nearly completely sterilized (no CFUs detected) with no outgrowth over 24 h (Fig. 4). Importantly, when the 5% GML Gel alone is plated onto an agar surface and then a comparable number of bacteria are spread across the surface, no antibacterial activity is exhibited, suggesting that the 5% GML Gel diffuses into and across the plate and loses activity.

**Fig 5:**
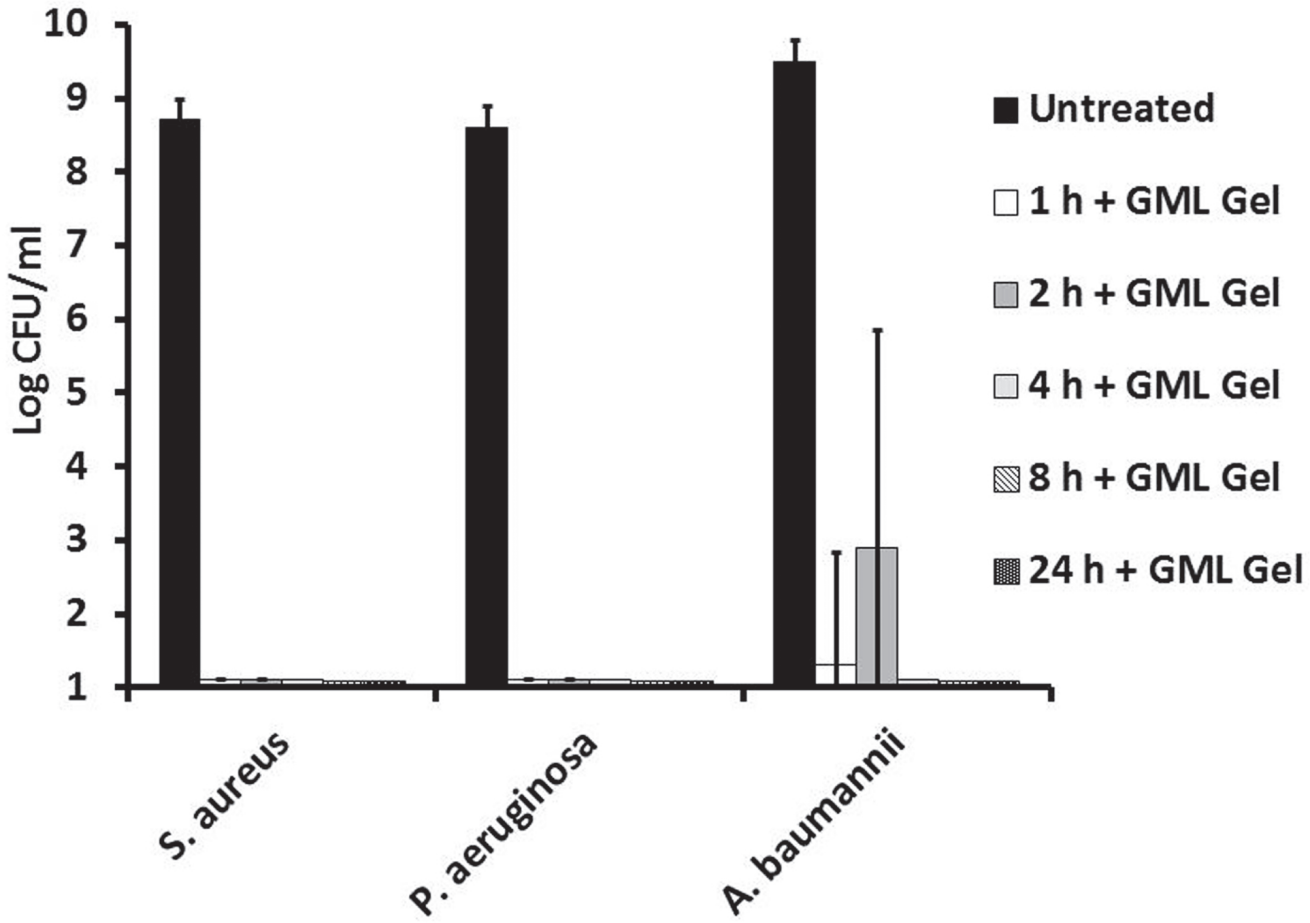
Anti-biofilm activity of 5% Glycerol Monolaurate Gel against *Staphylococcus aureus*, *Pseudomonas aeruginosa*, and *Acinetobacter baumannii*. Mature stationary biofilms of *Staphylococcus aureus*, *Pseudomonas aeruginosa*, and *Acinetobacter baumannii* were grown in 24-well microtiter plates in 7% CO_2_ at 37°C. After 48 h of growth, media was removed and replaced by 5% Glycerol Monolaurate Gel. At time points, wells were mixed vigorously and plate counts were taken for CFU from three independent replicates. Bars represent standard deviation.

Similarly, mature biofilms were formed in microtiter plates and treated with either the 5% GML Gel or the NA gel control in triplicate. At time points, 0.1 sterile swabs were taken and plated for CFUs. *P*. *aeruginosa* and *S*. *aureus* MN8 GML Gel-treated biofilms were completely sterilized by 1 h post-treatment, and *A*. *baumannii* biofilms were completely sterilized 4 h post-treatment (Fig. 5). NA vehicle alone (100%) sterilized *S*. *aureus* MN8 and *P aeruginosa* biofilms 2 h post-exposure and *A*. *baumannii* at 4 h (data not shown).

### GML reduces bacterial burden and inflammation in a New Zealand white rabbit surgical site infection model

To assess the potential of the 5% GML Gel *in vivo*, we developed a New Zealand white rabbit model for surgical site infections. Briefly, subcutaneous incisions were made in the flank of anesthetized rabbits and closed with 4 silk sutures. Sutures were inoculated with approximately 10^10^ bacteria, a clinically relevant concentration for contaminated skin. Furthermore, inoculum concentrations of 10^7^ or greater have demonstrated greater resistance to antibacterial and anti-biofilm agents in suture-associated infections [22]. After the inoculum was allowed to absorb, sutures were painted with either 5% GML Gel or PBS as a control. Rabbits were allowed to wake, and swabs were taken at designated time points to determine bacterial burden. At sacrifice, sutures were opened to examine underlying tissue inflammation.

**Fig 6:**
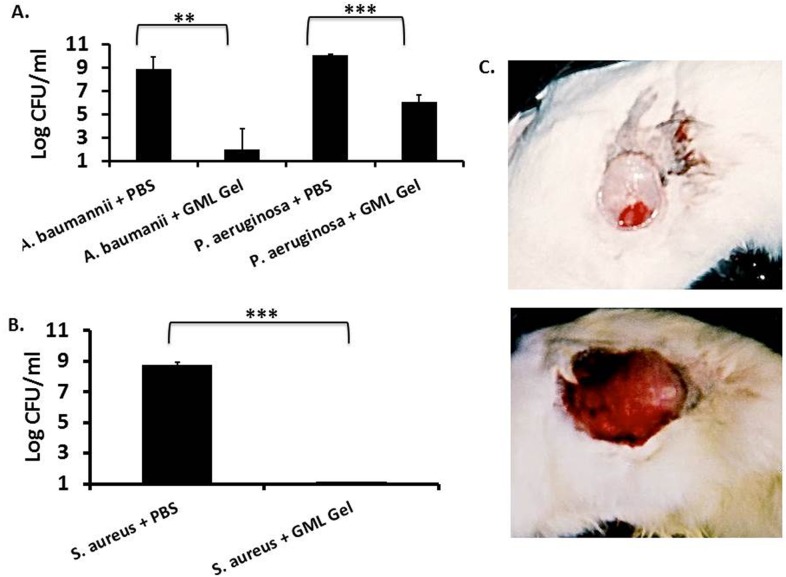
Effect of 5% Glycerol Monolaurate Gel treatment in New Zealand white rabbit surgical site infections. Four centimeter incisions were made in the flanks of New Zealand white rabbits, sutured closed, and inoculated with 10^10^ CFU of *Staphylococcus aureus*, *Pseudomonas aeruginosa*, or *Acinetobacter baumannii*. 5% Glycerol Monolaurate Gel or placebo was then liberally applied to the site. Three animals were used for treated and untreated groups per organism. Sites were assessed for bacterial burden and inflammation at the end of the experiment. At one hour post-treatment the bacterial burden was assessed for *Pseudomonas aeruginosa* and *Acinetobacter baumannii* infected groups (A), and at 24 h post-treatment, the bacterial burden was assessed for *Staphylococcus aureus* infected groups (B). Bars demonstrate standard deviation, ** and *** represent p ≤ 0.01 and p ≤ 0.001 respectively. A representative visual of inflammation between 5% Glycerol Monolaurate Gel (above) and PBS control (below) are pictured in C.

For the gram-negative bacteria exposed groups, the treatment and control rabbits cleared the organism by 24 h. In accordance with a large body of literature, we suspect gram-negative rods are less likely to colonize newly established wounds, such as burns and surgical site infections, without an immune-compromised host or marked wound deterioration by gram-positive bacteria [23–25]. Our model did not account for this, and therefore, we were only able to assess the bacterial burden one-hour post-treatment. For both *P*. *aeruginosa* and *A*. *baumannii*, the bacterial burden recovered from the site was significantly lower (p = 0.0003 and p = 0.004, respectively) for the GML Gel-treated group than the control group (Fig. 6A).

*S*. *aureus* MN8 was effectively able to colonize the surgical site, and we were able to examine bacterial burden of experimental and control groups at 24 h post-treatment. The untreated group averaged 5 x 10^8^ CFUs/swab, but for the GML Gel-treated group, no CFUs were recovered (Fig. 6B). Additionally, when we opened the surgical site and pulled back the skin to examine for subcutaneous inflammation, all three control rabbits had visible signs of inflamed skin and redness in the hide and tissue beneath the sutures. Comparatively, the GML-treated animals had no or minimal visible signs of inflammation within or under the sutures (Fig. 6C).

## Discussion

Our previous studies and others’ indicate that GML has a broad range of antimicrobial activity: it is a nonspecific signal transduction disruptant in gram-positive bacteria [12, 13], inhibits gram-positive exotoxin production at sub-growth-inhibitory concentrations [12–14], prevents enveloped virus penetration of mammalian cells [16], and reduces host inflammatory responses [15]. Furthermore, GMLhas no adverse effects on the host (nonhuman primates and humans) mucosal surface and microflora [17, 18], and microbial resistance does not develop, even after a year of passaging *S*. *aureus* on media containing a sub-inhibitory concentration of GML [13]. Taken together, these qualities indicate that GML has potential as a novel antimicrobial agent. However, until now, a limitation of GML’s activity is that it is not effective against gram-negative pathogens, particularly those with intact lipopolysaccharide layers [13].

We hypothesized that we could expand GML’s antibacterial range to include gram-negative organisms not susceptible to GML alone by combining GML delivery with a NA gel related to K-Y Warming. This delivery system previously was shown to be safe for chronic human and non-human primate use for up to six months and exhibit no deleterious effects on vaginal microflora [17, 18]. In the current study, we demonstrate that GML solubilized in the NA delivery vehicle has greater activity than GML alone. This GML Gel completely sterilizes planktonic and biofilm cultures of gram-positive and gram-negative pathogens. Furthermore, the gel reduces bacterial burden and tissue inflammation in a surgical site infection model in New Zealand white rabbits.

We postulate the efficacy of the GML Gel is attributable to many factors. First, we have demonstrated that the NA Gel has antimicrobial activity and that sub-growth-inhibitory concentrations of vehicle decreases the concentration of GML necessary for antibacterial activity. We speculate that this interaction is due in part to the membrane-disrupting effects of the vehicle, which many increase GML’s ability to penetrate the membrane. However, it is also plausible that the synergy results from the acidity of the vehicle (pH 4.5). Previously, we have observed that decreasing the pH of broth media increases the susceptibility of gram-negative pathogens, including *E*. *coli* and *P*. *aeruginosa*, to GML by up to 500-fold per each pH unit decrease [13]. We speculate this is due to protonation of the cell envelope and consequent repulsion of divalent cations from the cell surface, causing membrane destabilization. Because the NA vehicle is acidic, it is possible that this plays a role in the GML Gel’s antibacterial activity. Finally, GML is over 100-fold more soluble in the NA vehicle than in aqueous vehicles; perhaps, higher concentrations of GML are necessary to affect the growth of some pathogens. Taken together, these synergistic properties suggest multiple modes of action, likely limiting the target organism’s ability to develop resistance to the GML Gel. Additionally, we have previously attempted to induce microbial resistance to GML by weekly passage for one year on sub-inhibitory GML concentrations and then plating on inhibitory concentrations [13]. No resistance was seen. We hypothesized that the lack of resistance development to GML occurs because there are multiple GML targets in bacteria that result in inhibition of growth.

## References

[1] Bratzler DW, Hunt DR. The surgical infection prevention and surgical care improvement projects: national initiatives to improve outcomes for patients having surgery. Clin Infect Dis. 2006;43(3):322–30. 10.1086/505220 PubMed . 16804848

[2] National Nosocomial Infections Surveillance S. National Nosocomial Infections Surveillance (NNIS) System Report, data summary from January 1992 through June 2004, issued October 2004. Am J Infect Control. 2004;32(8):470–85. 10.1016/S0196655304005425 PubMed . 15573054

[3] Kirkland KB, Briggs JP, Trivette SL, Wilkinson WE, Sexton DJ. The impact of surgical-site infections in the 1990s: attributable mortality, excess length of hospitalization, and extra costs. Infect Control Hosp Epidemiol. 1999;20(11):725–30. 10.1086/501572 PubMed . 10580621

[4] Martone WJ, Nichols RL. Recognition, prevention, surveillance, and management of surgical site infections: introduction to the problem and symposium overview. Clin Infect Dis. 2001;33 Suppl 2:S67–8. 10.1086/321859 PubMed . 11486301

[5] Hidron AI, Edwards JR, Patel J, Horan TC, Sievert DM, Pollock DA, et al NHSN annual update: antimicrobial-resistant pathogens associated with healthcare-associated infections: annual summary of data reported to the National Healthcare Safety Network at the Centers for Disease Control and Prevention, 2006–2007. Infection control and hospital epidemiology: the official journal of the Society of Hospital Epidemiologists of America. 2008;29(11):996–1011. 10.1086/591861 PubMed . 18947320

[6] Kluytmans J, van Belkum A, Verbrugh H. Nasal carriage of Staphylococcus aureus: epidemiology, underlying mechanisms, and associated risks. Clinical microbiology reviews. 1997;10(3):505–20. Epub 1997/07/01. PubMed 9227864 10.1128/cmr.10.3.505 PMC172932

[7] Grimble SA, Magee TR, Galland RB. Methicillin resistant Staphylococcus aureus in patients undergoing major amputation. Eur J Vasc Endovasc Surg. 2001;22(3):215–8. 10.1053/ejvs.2001.1436 PubMed . 11506513

[8] Moran GJ, Krishnadasan A, Gorwitz RJ, Fosheim GE, McDougal LK, Carey RB, et al Methicillin-resistant S-aureus infections among patients in the emergency department. New England Journal of Medicine. 2006;355(7):666–74. 10.1056/Nejmoa055356 PubMed . 16914702

[9] Kirby AE, Garner K, Levin BR. The relative contributions of physical structure and cell density to the antibiotic susceptibility of bacteria in biofilms. Antimicrobial agents and chemotherapy. 2012;56(6):2967–75. 10.1128/AAC.06480-11 PubMed 22450987 PMC3370779

[10] Strateva T, Mitov I. Contribution of an arsenal of virulence factors to pathogenesis of Pseudomonas aeruginosa infections. Annals of Microbiology. 2011;61(4):717–32. 10.1007/S13213-011-0273-Y PubMed PMID: WOS:000296964900003.

[11] McConnell MJ, Actis L, Pachon J. Acinetobacter baumannii: human infections, factors contributing to pathogenesis and animal models. FEMS Microbiol Rev. 2013;37(2):130–55. 10.1111/j.1574-6976.2012.00344.x PubMed . 22568581

[12] Schlievert PM, Deringer JR, Kim MH, Projan SJ, Novick RP. Effect of glycerol monolaurate on bacterial growth and toxin production. Antimicrobial agents and chemotherapy. 1992;36(3):626–31. 1622174 10.1128/aac.36.3.626 PMC190568

[13] Schlievert PM, and Peterson M.L. Glycerol monolaurate antibacterial activity in broth and biofilm cultures. PloS one. 2012;7:e40350 10.1371/journal.pone.0040350 22808139 PMC3394780

[14] Lin YC, Schlievert PM, Anderson MJ, Fair CL, Schaefers MM, Muthyala R, et al Glycerol monolaurate and dodecylglycerol effects on Staphylococcus aureus and toxic shock syndrome toxin-1 in vitro and in vivo. PloS one. 2009;4(10):e7499 Epub 2009/10/20. 10.1371/journal.pone.0007499 PubMed 19838303 PMC2759527

[15] Peterson ML, Schlievert PM. Glycerol monolaurate inhibits the effects of gram-positive select agents on eukaryotic cells. Biochemistry. 2006;45(7):2387–97. PubMed . 16475828 10.1021/bi051992u PMC2553893

[16] Li Q, Estes JD, Schlievert PM, Duan L, Brosnahan AJ, Southern PJ, et al Glycerol monolaurate prevents mucosal SIV transmission. Nature. 2009;458(7241):1034–8. Epub 2009/03/06. doi: nature07831 [pii] 10.1038/nature07831 PubMed . 19262509 PMC2785041

[17] Strandberg KL, Peterson ML, Lin YC, Pack MC, Chase DJ, Schlievert PM. Glycerol monolaurate inhibits Candida and Gardnerella vaginalis in vitro and in vivo but not Lactobacillus. Antimicrobial agents and chemotherapy. 2010;54(2):597–601. Epub 2009/12/17. doi: AAC.01151-09 [pii] 10.1128/AAC.01151-09 PubMed 20008774 PMC2812150

[18] Schlievert PM, Strandberg KL, Brosnahan AJ, Peterson ML, Pambuccian SE, Nephew KR, et al. Glycerol Monolaurate Does Not Alter Rhesus Macaque (Macaca mulatta) Vaginal Lactobacilli and Is Safe for Chronic Use. Antimicrobial agents and chemotherapy. 2008. PubMed . 18838587 10.1128/AAC.00989-08 PMC2592867

[19] Kwasny SM, Opperman TJ. Static biofilm cultures of Gram-positive pathogens grown in a microtiter format used for anti-biofilm drug discovery. Curr Protoc Pharmacol. 2010;Chapter 13:Unit 13A 8. doi: 10.1002/0471141755.ph13a08s50 PubMed 22294365 PMC3272335

[20] Park JH, Lee JH, Cho MH, Herzberg M, Lee J. Acceleration of protease effect on Staphylococcus aureus biofilm dispersal. FEMS Microbiol Lett. 2012;335(1):31–8. 10.1111/j.1574-6968.2012.02635.x PubMed . 22784033

[21] Xiong Y, Liu Y. Importance of extracellular proteins in maintaining structural integrity of aerobic granules. Colloids Surf B Biointerfaces. 2013;112:435–40. 10.1016/j.colsurfb.2013.07.060 PubMed . 24036627

[22] Hess DJ, Henry-Stanley MJ, Wells CL. Interplay of antibiotics and bacterial inoculum on suture-associated biofilms. J Surg Res. 2012;177(2):334–40. 10.1016/j.jss.2012.04.040 PubMed 22682712 PMC3498097

[23] Trinh JV, Chen LF, Sexton DJ, Anderson DJ. Risk factors for gram-negative bacterial surgical site infection: do allergies to antibiotics increase risk? Infect Control Hosp Epidemiol. 2009;30(5):440–6. 10.1086/596612 PubMed . 19317629

[24] Pruitt BA Jr., McManus AT. Opportunistic infections in severely burned patients. Am J Med. 1984;76(3A):146–54. PubMed . 6369976 10.1016/0002-9343(84)90334-6

[25] Church D, Elsayed S, Reid O, Winston B, Lindsay R. Burn wound infections. Clin Microbiol Rev. 2006;19(2):403–34. 10.1128/CMR.19.2.403-434.2006 PubMed 16614255 PMC1471990

